# The benefit of knowledge: postural response modulation by foreknowledge of equilibrium perturbation in an upper limb task

**DOI:** 10.1007/s00421-023-05323-z

**Published:** 2023-09-27

**Authors:** Juan M. Castellote, Markus Kofler, Andreas Mayr

**Affiliations:** 1 Radiology, Rehabilitation and Physiotherapy Department, Faculty of Medicine, Universidad Complutense, Madrid, Spain; 2 Department of Neurology, Hochzirl Hospital, Zirl, Austria

**Keywords:** Motor program, Startle reaction, StartReact effect, Kinematic stimulus, Proprioception

## Abstract

For whole-body sway patterns, a compound motor response following an external stimulus may comprise reflexes, postural adjustments (anticipatory or compensatory), and voluntary muscular activity. Responses to equilibrium destabilization may depend on both motor set and a subject`s expectation of the disturbing stimulus. To disentangle these influences on lower limb responses, we studied a model in which subjects (*n* = 14) were suspended in the air, without foot support, and performed a fast unilateral wrist extension (WE) in response to a passive knee flexion (KF) delivered by a robot. To characterize the responses, electromyographic activity of rectus femoris and reactive leg torque was obtained bilaterally in a series of trials, with or without the requirement of WE (motor set), and/or beforehand information about the upcoming velocity of KF (subject`s expectation). Some fast-velocity trials resulted in StartReact responses, which were used to subclassify leg responses. When subjects were uninformed about the upcoming KF, large rectus femoris responses concurred with a postural reaction in conditions without motor task, and with both postural reaction and postural adjustment when WE was required. WE in response to a low-volume acoustic signal elicited no postural adjustments. When subjects were informed about KF velocity and had to perform WE, large rectus femoris responses corresponded to anticipatory postural adjustment rather than postural reaction. In conclusion, when subjects are suspended in the air and have to respond with WE, the prepared motor set includes anticipatory postural adjustments if KF velocity is known, and additional postural reactions if KF velocity is unknown.

## Introduction

Muscular activity is controlled by central nervous system commands, which can either be generated at free will, or which may derive non-voluntarily and reflexively in response to an external stimulus. Extensive research is available in the field of motor responses during whole-body human activities such as sit-to-stand maneuvers, gait, or localized adjustments for tool manipulation with upper limbs (Morris et al. 2001; Baker 2018; Vaidya et al. 2017; da Costa et al. 2010; Santello et al. 2016; Reissner et al. 2019). For whole-body sway patterns, a compound motor response following an external stimulus may comprise reflexes, postural adjustments (anticipatory or compensatory), and voluntary muscular activity.

To investigate whole-body postural control, particularly equilibrium, subjects are usually studied while standing, with the lower limbs fulfilling the main role in maintaining stable posture to provide the desired framework for subjects to perform voluntary acts, mainly with the hands. Voluntary upper limb movements are preceded by anticipatory postural adjustments (APA), which can be recorded in the lower limbs (Massion et al. 1999; Delafontaine et al. 2019). They appear as part of the motor reaction at latencies, which may coincide with volitional activity (later than 100 ms after an imperative signal, IS, in reaction time paradigm tasks). Furthermore, compensatory postural adjustments (CPA) may appear in the lower limbs following volitional upper limb movement. Both APA and CPA seem to act in synergy to achieve a subject’s rapid stabilization after perturbation. In addition, if a mechanical disturbance destabilizes the standing subject, additional muscle responses are evoked in the lower limbs (presumably by sudden stretch) within 100 ms corresponding to what has been described as the short latency reflex (SLR), followed by the long latency reflex (LLR) (Santos et al. 2010a; Vedula et al. 2010; Helm et al. 2019).

However, conditions in which the legs do not support the verticalized subject have scarcely been studied. This is the case when subjects are in a suspended position, the trunk being secured by a harness (e.g., housepainters, construction workers, carpenters, and tree-trimmers). In these situations, equilibrium is maintained with the trunk rather than with the legs. The center of support may then be transferred from the legs mainly to the trunk, particularly when sitting in an unstable posture, or when hanging from roofs close to walls, or occasionally when shifting weight to the arms, e.g., during over-head drilling. In these circumstances the fulcrum for the action is not the ankle, as it is in standing. As a consequence, the inverse pendulum model from the feet to the moving segment, usually hands and arms, is no longer valid, thus the relative contribution of the lower limbs to the task is unknown in those circumstances in which the trunk takes over equilibrium control. Some similarities can also be found in neurorehabilitation, when for certain assessments or therapeutic procedures, patients are lifted from the ground and secured at waist and trunk level by a harness, rendering them suspended in the air with little or no weight-support on their legs. This is the case when patients following stroke or spinal cord injury are assessed for lower limb spasticity, or undergo gait training with the aid of supporting harnesses and, in recent decades, with exoskeletons (Mayr et al. 2007, 2019; Mirbagheri et al. 2012). Harnesses mainly reduce the weight supported by the legs but avoid or modify the fulcrum function of the legs. Exoskeletons may provide leg stability and support active movement in paretic legs.

Consequently in suspended subjects, fast postural adjustments and local reactions in leg muscles following remote voluntary movements (e.g., reaching and grasping) may be different from those previously described for standing upright on firm ground (Jacobs & Horak 2007; Horak et al. 1989; Diener et al. 1988). The contribution of each component to this modulation may depend on the “preparedness” of the subject to react, i.e., on the degree of mental expectation of the stimulus. Various authors have described the participation of each of these processes for the lower limbs in health and disease (Leukel et al. 2009; Rabita et al. 2005; Lamontagne et al. 1998; Mrachacz-Kersting et al. 2004, 2006) as well as following upper limb movements (Pruszynski et al. 2008; Pruszynski and Scott 2012).

The so-called StartReact effect (Valls‐Solé et al. 1999) might be an experimental procedure serving to uncover these adjustments and reactions in subjects suspended in the air. The StartReact effect was previously explored for fast hand reactions in such a condition (Castellote et al. 2017), but the participation of lower limbs to the upper limb motor program has not been investigated. The StartReact effect reveals a pre-programmed motor task by means of accelerated execution in situations where the study paradigm contains a surprising component, eliciting a startle reaction (reflex). This effect has been described for different activities, e.g., sit-to-stand (Queralt et al. 2008), saccades (Castellote et al. 2007), wrist extension (Maslovat et al. 2014; Castellote et al. 2017), and eye opening (Valls‐Solé et al. 2021). To further characterize the StartReact effect, a weak sensory stimulus can be applied prior to the strong reflex-eliciting stimulus to suppress the reflex component without affecting response acceleration (Valls-Solé et al. 2005) (Castellote et al. 2017). This suppression of reflex magnitude is termed prepulse inhibition and entails the pedunculopontine nucleus (Garcia-Rill et al. 2019).

The goal of the present study was to advance current knowledge on motor preparation by exploring a model in which subjects are suspended upright in the air without foot support, performing a fast voluntary response with one hand (wrist extension, WE) upon a fast mechanical stimulus delivered to one leg (knee flexion, KF), theoretically destabilizing the subject and in some occasions being of startling intensity (fast KF) (Castellote et al. 2017). We expected a different response when subjects are suspended in the air without leg support, as opposed to known reactions when standing on firm ground (Santos et al. 2010a; MacKinnon et al. 2007; Fiset and McFadyen 2020; Liaw et al. 2021). The present study complements our previous report of fast hand reactions obtained in a subset of the same subjects (Castellote et al. 2017) and now focuses on the influence of the subjects’ expectation and preparedness to perform an upper limb motor task on associated motor reactions in the legs. We hypothesized that reflexes would increase with intensity (i.e., angular velocity of KF) and that responses would be altered when the legs participate in the hand motor task. The results may shed light on whether there is central pre-activation of leg muscles when suspended in the air. If lower limb activation is indeed required for the execution of the hand motor program, we would expect differences in APA or CPA.

## Methods

### Subjects

Fourteen self-reportedly healthy participants (8 females, 6 males, age 27–52 years) took part in the experiment. Subjects were excluded if they reported any personal history of conditions which could cause disturbance of equilibrium or motor control, repeated stumbling, or if any related symptoms or signs were noticed in a brief neurological exam. All participants gave signed informed consent for the study, which was approved by the local Institutional Review Board.

### Setup

Participants were placed in an electromechanical gait robot (Lokomat, DIH/Hocoma, Switzerland) with each lower limb strapped to an exoskeleton system adjusted to individual height and leg length. Participants were suspended in the air during trial periods by a harness around the torso and pelvis by means of an over-head body weight-support system with deflection pulleys. Thus, the legs hung freely, only attached to the orthoses of the robot. Handrails at waist level allowed supporting if necessary, and a horizontal band in front of the participant enabled resting the hands and forearms as previously described (Castellote et al. 2017). Briefly, the wrist was held in a neutral position concerning flexion–extension and the hand in slight pronation. The Lokomat was used to produce passive KF at pre-set angular velocities acting as IS in a simple reaction time task. In most trials, participants were asked to perform a fast right WE as soon as they perceived the IS in the leg. In few selected trials, an electrical stimulus generated with a Digitimer D180A (0.1 ms duration, 1.5 times perception threshold) was delivered through ring electrodes placed on the left index finger, 100 ms preceding the IS, to induce prepulse inhibition. The resistive leg torques associated with the induced movement were recorded with built-in force transducers of the Lokomat exoskeleton and stored for off-line analysis. Electromyographic (EMG) responses were recorded with an electrodiagnostic system (Viking IV, Natus-Nicolet Biomedical, Madison, Wisconsin) synchronized with the Lokomat. The sweep was triggered by the Lokomat beginning from a starting point with completely extended knees. Subsequent knee extension back to the starting point was always performed at a low velocity (less than 10°/s). The electrodiagnostic equipment recorded single sweeps of 4 s, including a 900 ms pre-stimulus delay. EMG signals were obtained from the left and right rectus femoris muscles (RF) with pairs of surface electrodes to quantify stretch-related muscle activity. RF was chosen because it is a postural muscle, easy to record from, and the main source of resistive torques following KF. EMG activity related to WE was recorded from right extensor carpi radialis muscle (ECR). As the IS can be a source of a startle reaction, surface EMG activity was recorded from the right orbicularis oculi (OOc) and sternocleidomastoid muscles (SCM), which are considered “startle indicator muscles” (Carlsen et al. 2011; Forgaard et al. 2018). Filter settings were 10–10,000 Hz.

### Procedure and sequence of trials

**Fig. 1:**
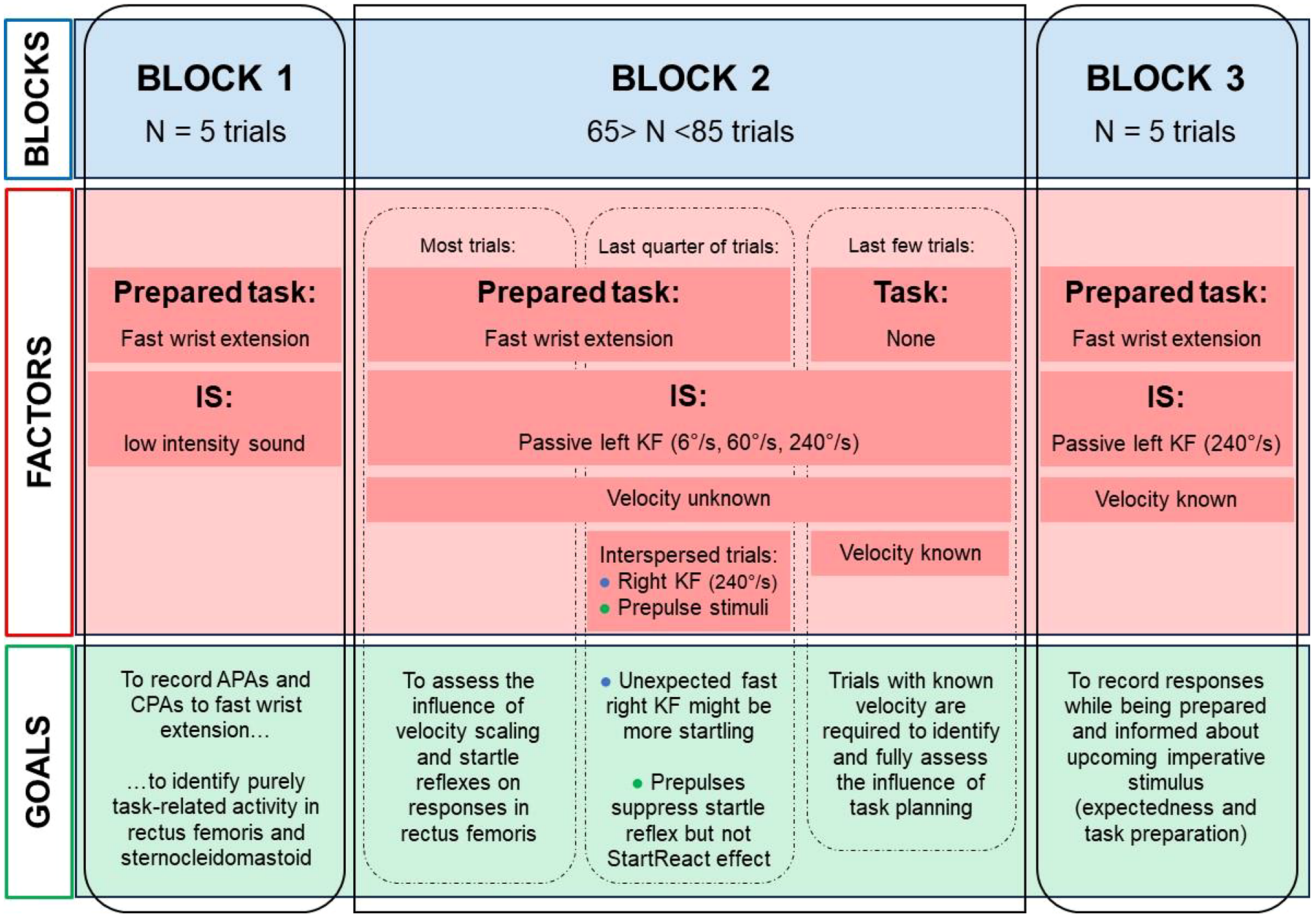
Flow chart that presents the procedure and sequences of blocks, factors, and goals

Participants were instructed that there would be a series of trials, in which (with few specific exceptions) they should perform a fast WE as soon as they perceived the IS (i.e., passive KF induced by the robot). They were instructed not to perform any resistance or assistance to the leg movement and to relax during all experiments. Each trial included a verbal warning for the subject to be prepared (“ready!”), the IS delivered 1–3 s following the warning signal, and recording of the participant’s responses. Consecutive trials were separated by a minimum of 1 min, for the system to again reach the starting position, to provide time for subjects to relax, and to avoid influence of one trial upon the subsequent one. The study included established stops and lowering of the participants from the electromechanical device at certain times. In addition, participants could be lowered to standing on the floor, or, if desired, detached from the system, at any time if they felt uncomfortable. There was a predetermined workflow of trials, grouped in three blocks (Fig. 1).

Block 1 allowed participants to get accustomed to the suspension in the system. It was composed of five trials to record APAs and CPAs to fast WE. Only in this block a low intensity tone burst (60 dB nHL, 500 Hz, 10 ms duration) was used as IS instead of passive KF. The trials permitted depicting the participant’s movement pattern employed during brisk WE while hanging suspended in the harness. Specifically, the goal was to ascertain whether any EMG activity in RF or SCM was associated with the task which could interfere with analysis of responses in the subsequent trials.

**Fig. 2:**
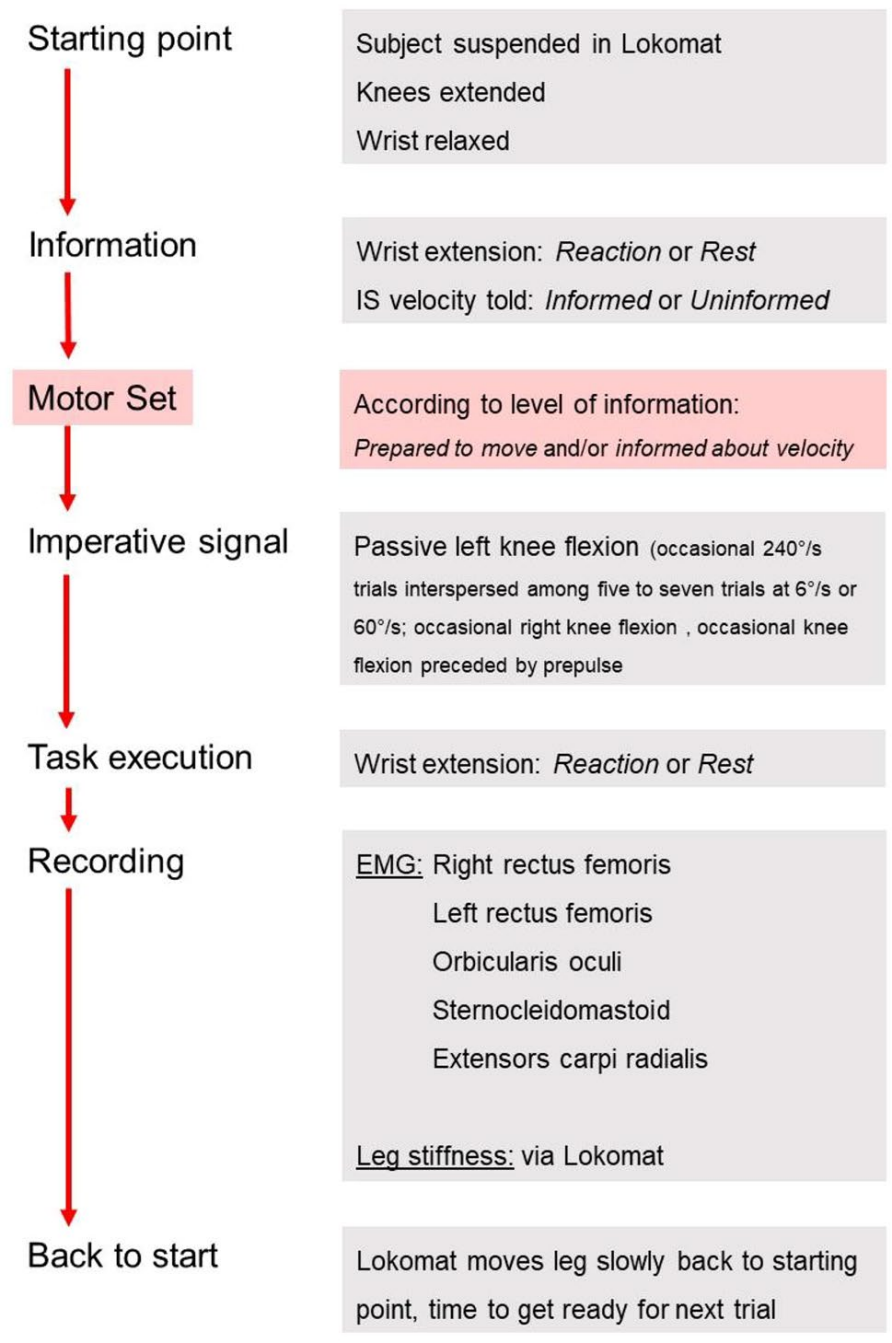
Sequence of events for a representative trial of the main block (Block 2)

In blocks 2 and 3, participants were instructed to respond with a fast WE in most trials upon perceiving the IS. Trials with fast angular velocity of left KF (240°/s) interspersed with trials at slower velocities (6°/s, 60°/s), or right KF (240°/s) during the last quarter of block 2, were applied in pseudorandom order (Fig. 2). The Lokomat established a total angular displacement of 80° for trials at 240°/s and 60°/s. For a 6°/s trial, the established excursion was 40°. To avoid rapid habituation, each of the 240°/s trials was interspersed with at least 5 trials at 6°/s, and occasional trials at 60°/s. In most trials with few specific exceptions in block 2, subjects were not informed beforehand about the type of upcoming stimuli. We expected the occasional presence of startle reflexes in 240°/s trials of block 2, thereby modifying WE similar to the previously described StartReact effect in other settings (Queralt et al. 2008; Castellote et al. 2007; Maslovat et al. 2014), and in fact, they were occasionally present (Castellote et al. 2017). During the last fourth of block 2, few 240°/s trials were performed either applying a prepulse, to suppress the startle reflex magnitude without affecting the StartReact effect (Valls-Solé et al. 2005; Castellote et al. 2017), or applying the passive KF (240°/s) unexpectedly in the opposite leg to render the stimulus more surprising. Block 2 ended with a few trials in which participants were asked not to perform any WE when the IS was delivered. In some of these trials without WE, participants knew the angular velocity of KF, in others not. The number of trials per subject were pre-defined as follows for each velocity: for left KF, 12 for 6°/s, 10 for 60°/s, 6 for 240°/s, 3 for 240°/s with prepulse, 6 for the final trials without WE; for right KF, 3 for 240°/s. OOc and SCM activity was visually monitored online, confirming at least three recordings with startle reflexes and three without for left KF 240°/s trials. If needed, additional 240°/s trials were recorded, each interspersed with at least 5 trials at lower angular velocities, resulting in a total of 65–85 trials per subject.

Block 3 included 5 left KF 240°/s trials as IS for WE, in which participants were explicitly informed about the upcoming velocity.

### Data classification

Trials with left KF as IS were classified according to three factors: IS INFORMATION (2 levels: *Informed*, *Uninformed*), IS VELOCITY (3 levels: 6°/s, 60°/s, 240°/s), and WE TASK (2 levels: *Reaction*, *Rest*). Additional classifications were done for those trials where right KF was the IS (“OPP” for opposite leg), for those that included a prepulse (“PP”, left index finger stimulus present), and for those that had startle signs, i.e., either startle reflexes or a StartReact effect (“SR”, appropriate EMG activity in OOc and/or SCM and acceleration of WE based on ECR EMG latency) as previously described in detail (Castellote et al. 2017).

### Data processing and analysis

EMG data were analyzed with the built-in software of the electrodiagnostic equipment. Analysis of torque data for the different conditions was performed using the Lokomat’s measurement tool for analysis. The start of the IS, i.e., movement onset in the leg established with an accelerometer attached to the tibial crest, was the reference time for all response latencies.

To assess whether EMG responses might be modified dependent on differences in background EMG (Scheirs and Brunia 1986; Ogiso et al. 2002) rather than differences in any of the factors, integrated EMG (iEMG) of RF in the passively moved leg was measured as area-under-the-curve (henceforth “area”) during a 50 ms baseline period preceding movement onset for later comparison.

For a 240°/s trial (Fig. 1), the RF reaction to the IS showed EMG activity that could correspond to either reflexes or adjustments, and was classified based on latencies in three time windows in accordance with previous reports (Dietz et al. 1987; Bergui et al. 1992; Mrachacz-Kersting and Sinkjaer 2003; Mrachacz-Kersting et al. 2004).

First, SLR period, starting at movement onset and lasting 50 ms. SLR onset was determined by visual inspection of the EMG trace as described elsewhere (Mrachacz-Kersting et al. 2006). SLR magnitude was measured as area during a 20-ms time window beginning from SLR onset.

Second, LLR period, lasting from 50 to 100 ms after movement onset. Concurring with previous literature (Mrachacz-Kersting et al. 2004; Bergmann et al. 2013; McPherson et al. 2018), SLR and LLR often merged, which precluded exact measurement of LLR onset. Therefore, we decided to measure LLR area in a fixed 50-ms time window, from 50 to 100 ms after movement onset.

Third, postural adjustment (PA) period, lasting from 100 to 200 ms after movement onset. Activities that start around 120 ms following a perturbation contain volitional responses (Lee and Tatton 1975). In this case, they correspond to PA in the leg as a consequence of WE and/or disequilibrium. Visual inspection revealed that the LLR often merged with the early PA component. Therefore, PA area but not latency was measured during a 100-ms time window from 100 to 200 ms after movement onset.

**Fig. 3:**
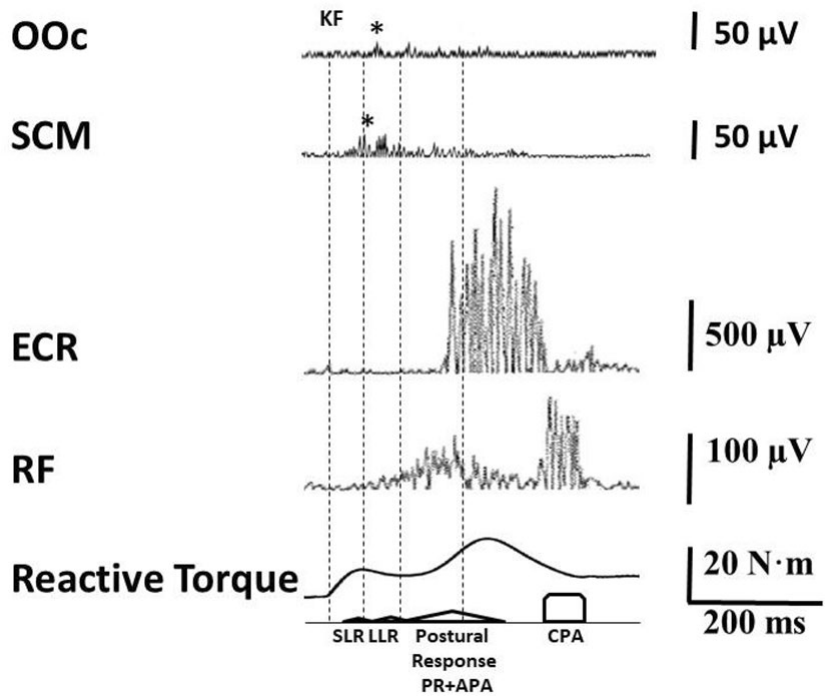
Representative trial from one subject depicting electromyographic (EMG) responses from orbicularis oculi (OOc), sternocleidomastoid (SCM), extensor carpi radialis (ECR), and rectus femoris (RF) muscles, as well as reactive torque of the leg in the experimental condition *Uninformed-Reaction*_SR_. The StartReact effect is supported by the presence of OOc and SCM responses (marked with asterisks: *) as well as an early ECR response (as compared to trials without concomitant responses in OOc or SCM). The RF trace shows the response to 240°/s knee flexion (KF) with activity starting at a latency corresponding to the short latency reflex (SLR), merging with an EMG burst around 200 ms, reflecting both long latency reflex (LLR) and postural response, that may include a postural reaction (PR) to KF and an anticipatory postural adjustment (APA) to wrist extension. A later EMG burst around 350–400 ms reflects the compensatory postural adjustment (CPA) to wrist extension. The torque reaction starts immediately following KF, reflecting the mechanical resistance of the leg to stretching, followed by two peaks corresponding to the subject's reflex and reactive responses

For all trials irrespective of angular velocity, stiffness was analyzed by means of the Lokomat measurement tool. For each trial, the resistive force obtained from the moved leg against the displacement was recorded during the entire movement period and expressed as torque-time relationship. For a 240°/s trial (Fig. 3), the reactive force showed two peaks separated by a trough approximately 150–200 ms after movement onset. We measured latencies from movement onset to each peak and peak amplitudes for each trial. Furthermore, we estimated reflexive and postural response (PR) components according to Mrachacz-Kersting and Sinkjaer (2003).

Data for each variable and factor were averaged per subject. These individual mean values were used to calculate group mean values and standard deviation for each variable and condition. Inferential analysis was performed for leg responses, particularly for 240°/s trials: SLR latency and amount of iEMG activity for the referred periods: pre-movement background, SLR, LLR, and PA. The analysis included peak torque latencies and peak torque amplitudes. Statistical significance was considered at *p* < 0.05.

Thus, the following comparisons were made:

- a three-way ANOVA with factors “IS INFORMATION”, “IS VELOCITY”, and "WE TASK” was performed to determine the influence of pre-movement background EMG on the ensuing reflex responses and reactions.
- a one-way ANOVA with factor “IS VELOCITY” was performed to compare the influence of velocity scaling on iEMG and torque responses in trials without information about the IS (factor “IS INFORMATION”: *Uninformed*) and with WE requested (factor “WE TASK”: *Reaction*).
- a two-factor ANOVA with factor “IS INFORMATION” and with factor "WE TASK” was performed in 240°/s trials only to elaborate how reaction strategies depended on the “preparedness” of participants and how RF responses were modulated in the absence of a StartReact effect.

## Results

### General remarks

All subjects performed the complete set of trials without difficulty. Two participants asked for additional interruption while suspended in the harness and for lowering from the system during the second block. In blocks 2 and 3, some trials were excluded from analysis (less than 5%/subject) due to signal interference from the robotic system. In block 1, WE in response to an acoustic stimulus while being suspended in the harness elicited EMG activity in ECR and RF with latencies of 230 ± 54 ms and 364 ± 60 ms following the IS, respectively. RF activity appeared consistently in all subjects, never preceded WE, and presumably contributed to compensate for the brisk WE. Few trials showed SCM activity with a latency of 200 ± 20 ms, presumably contributing to head stabilization while performing WE.

For both blocks 2 and 3, an ANOVA was performed to ascertain a similar background EMG activity before task execution in both RF and ECR among all three factors, i.e., IS INFORMATION (*Informed*, *Uninformed*), IS VELOCITY (6, 60, 240°/s), and WE TASK (*Reaction*, *Rest*) for each subject (RF: *F*_6,91_ = 0.04; *p* > 0.05; ECR: *F*_6,91_ = 0.12; *p* > 0.05).

Trials with KF at 240°/s (ipsi- or contralateral leg; both with and without prepulses), in which WE was required, were subclassified according to the presence or absence of responses in OOc and SCM at typical startle latencies (Valls-Solé et al. 1999; Carlsen et al. 2007) and of a StartReact effect in ECR. Accordingly, these trials were named: *Uninformed-Reaction*_SR,_ *Uninformed-Reaction*_OPP-SR_ and *Uninformed-Reaction*_PP-SR_. The remaining trials in the left leg without concomitant startle signs were classified as *Uninformed-Reaction*_no SR_.

Figure 3 depicts a representative *Uninformed-Reaction*_SR_ trial at 240°/s. During the SLR time window, EMG activity in RF emerges from ongoing low background activity and continues rising with a burst around 100–200 ms, presumably corresponding to both LLR and PA, followed by another peak around 300–400 ms, presumably reflecting additional CPA RF activity following WE. Torque activity in the passively moved leg begins immediately after the IS, resulting in a curve with two peaks and a trough around 150–200 ms. OOc and SCM activity is present at latencies concurring with a startle reflex.

### Influence of speed gain scaling

**Fig. 4:**
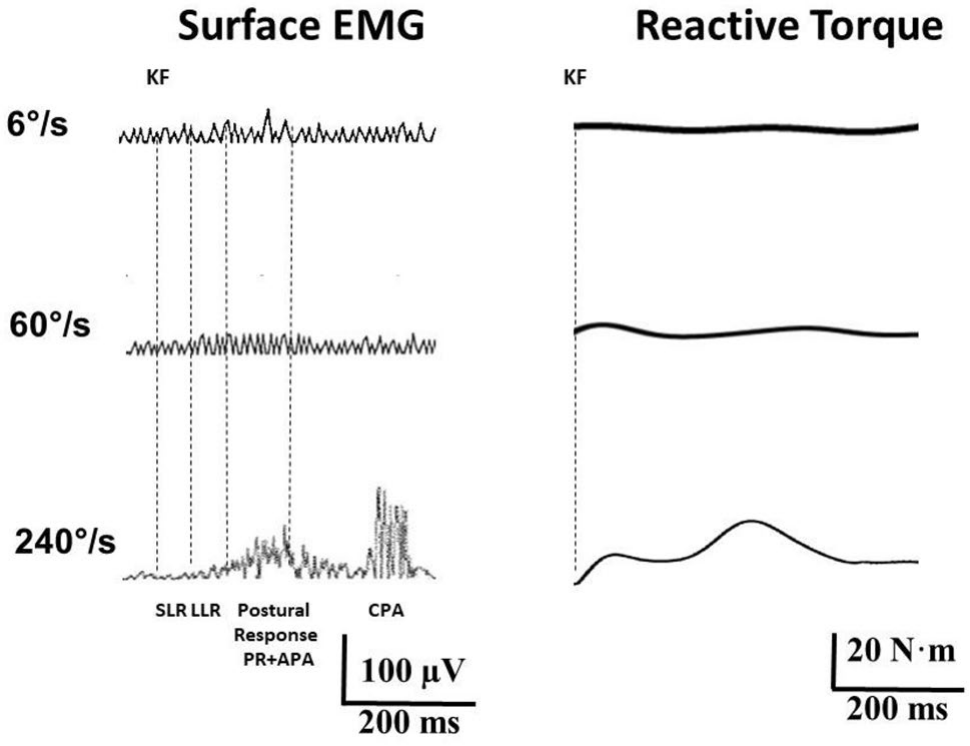
Influence of speed gain scaling. Representative trials depicting surface EMG responses from rectus femoris muscle and reactive torque of the extended leg to knee flexion in the condition *Uninformed-Reaction* at 6°/s, 60°/s and 240°/s angular velocities. The EMG traces show similar activity for the three velocities during the short latency reflex (SLR) and long latency reflex (LLR) periods. However, during the Postural Response period, larger EMG activity is present in the 240°/s trial as compared to the 6°/s and 60°/s trials. The 240°/s trial in fact corresponds to an *Uninformed-Reaction*_SR_ trial. Torque reaction is minimal at both 6°/s and 60°/s but shows two distinct peaks at 240°/s. Acronyms as in Fig. 1

The influence of different angular velocities used as IS was examined in *Uninformed-Reaction* trials. Compared to 240°/s *Uninformed-Reaction* trials, EMG activity in ECR appeared clearly later in 6°/s and 60°/s *Uninformed-Reaction* trials and was not accompanied by startle-related EMG activity in OOc and SCM (Fig. 4). Occasional RF responses during the SLR period did not differ in onset latency (*F*_2,39_ = 3.53; *p* > 0.05) and area (*F*_2,39_ = 2.11; *p* > 0.05) among the three angular velocities. EMG activity in RF was present in most 6°/s and 60°/s trials during both LLR and PA periods, but EMG area was significantly smaller in 6°/s and 60°/s than in 240°/s trials (LLR: *F*_2,39_ = 4.11; *p* < 0.05; PA: *F*_2,39_ = 5.58; *p* < 0.01). WE was regularly followed by CPA RF activity in 240°/s trials, but not always in 6°/s and 60°/s trials.

Torque was lower in 6°/s and 60°/s as compared to 240°/s trials, and the respective torque/time curve resulted in a dome-like shape lacking clear peaks. In contrast, 240°/s trials showed two distinct peaks at latencies of 135 ± 47 ms and 315 ± 30 ms with respective amplitudes of 10 ± 3 Nm and 12 ± 4 Nm.

### Influence of task planning

The participant`s response depends not only on IS velocity, but also on their motor strategy, the planning of the decided response. In the present paradigm this is fed by two factors: information about upcoming IS velocity and preparedness to react or to rest.

To estimate how the decision taken to act according to information about the upcoming IS velocity modulates WE, we compared *Informed-Reaction* and *Uninformed-Reaction* trials, yielding significantly shorter ECR latency and lower ECR area in *Informed-Reaction* trials (*p* < 0.05 each, paired t-test).

To estimate how knowledge about upcoming IS velocity and preparation of a motor task modulates reactions in RF, we compared EMG activity in RF in 240°/s trials without StartReact effect (*Uninformed-Reaction*, *Uninformed-Rest*, *Informed-Reaction*, *Informed-Rest*).

**Fig. 5:**
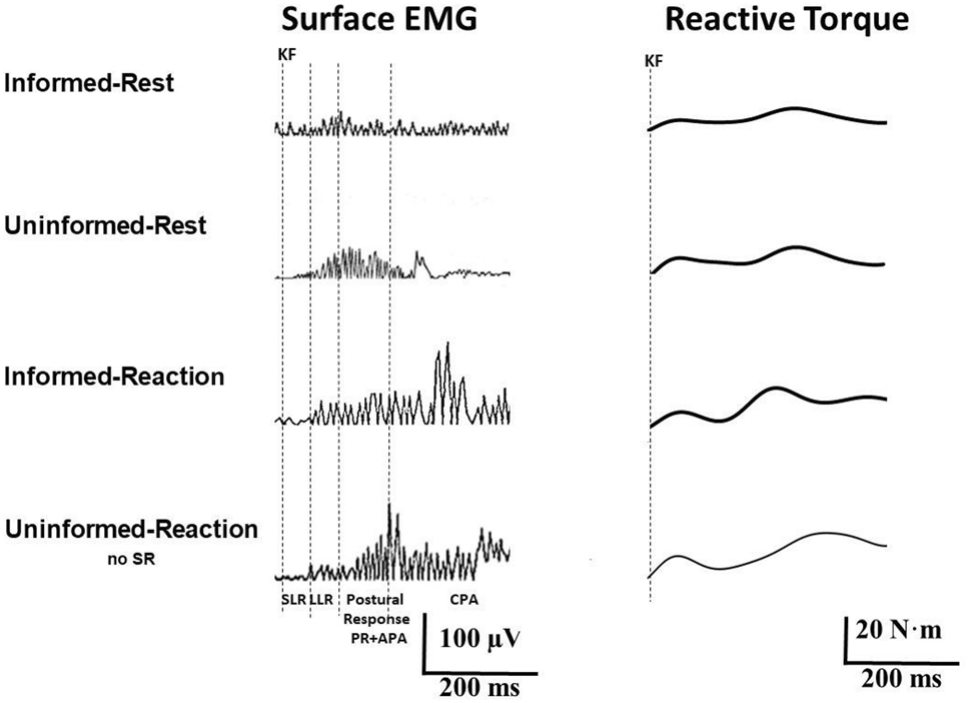
Influence of task planning. Surface EMG from rectus femoris muscle and reactive torques of the extended leg to knee flexion for representative 240°/s trials in the conditions *Informed-Rest, Uninformed-Rest*, *Informed-Reaction*, and *Uninformed-Reaction*. Note the larger EMG activity in the LLR and Postural Response periods in *Uninformed* vs *Informed* trials and in *Reaction* vs *Rest* trials. Torque peak amplitudes are larger for *Uninformed* trials as compared to *Informed* trials (both peaks), and in *Reaction* trials vs *Rest* trials (second peak). Acronyms as in Fig. 1

During the SLR period, advance knowledge about upcoming IS velocity did not significantly affect SLR latency (*F*_1,52_ = 1.77; *p* > 0.05) nor SLR area (*F*_1,52_ = 0.12; *p* > 0.05), and neither did the performance of WE (SLR latency: *F*_1,52_ = 0.01; *p* > 0.05; SLR area: *F*_1,52_ = 0.63; *p* > 0.05) (Fig. 5).

During the LLR period, advance knowledge about upcoming IS velocity resulted in significantly larger LLR area in *Uninformed* vs *Informed* trials (*F*_1,52_ = 7.52; *p* < 0.01). The requirement of WE resulted also in larger LLR area (*F*_1,52_ = 4.05; *p* < 0.05) in *Reaction* vs *Rest* trials without significant INFORMATION × TASK interaction (*F*_1,52_ = 0.4; *p* > 0.05) (Fig. 5).

Likewise, during the PA period, advance knowledge about IS velocity resulted in significantly larger PA area in *Uninformed* vs *Informed* trials (*F*_1,52_ = 34.63; *p* < 0.001), and the performance of WE gave rise to larger PA area in *Reaction* vs *Rest* trials (*F*_1,52_ = 6.71; *p* < 0.05) without INFORMATION × TASK interaction (*F*_1,52_ = 0.01; *p* > 0.05) (Fig. 5).

Neither advance knowledge about IS velocity nor the requirement of WE influenced latencies of the first or second peak in torque measurements in the 240°/s trials without startle reflexes (*Informed* vs *Uninformed*, first peak: *F*_1,52_ = 0.01; *p* > 0.05; second peak: *F*_1,52_ = 0.28; *p* > 0.05; *Reaction* vs *Rest*, first peak: *F*_1,52_ = 0.06; *p* = 0.7; second peak: *F*_1,52_ = 0.27; *p* = 0.6) (Fig. 5).

**Fig. 6:**
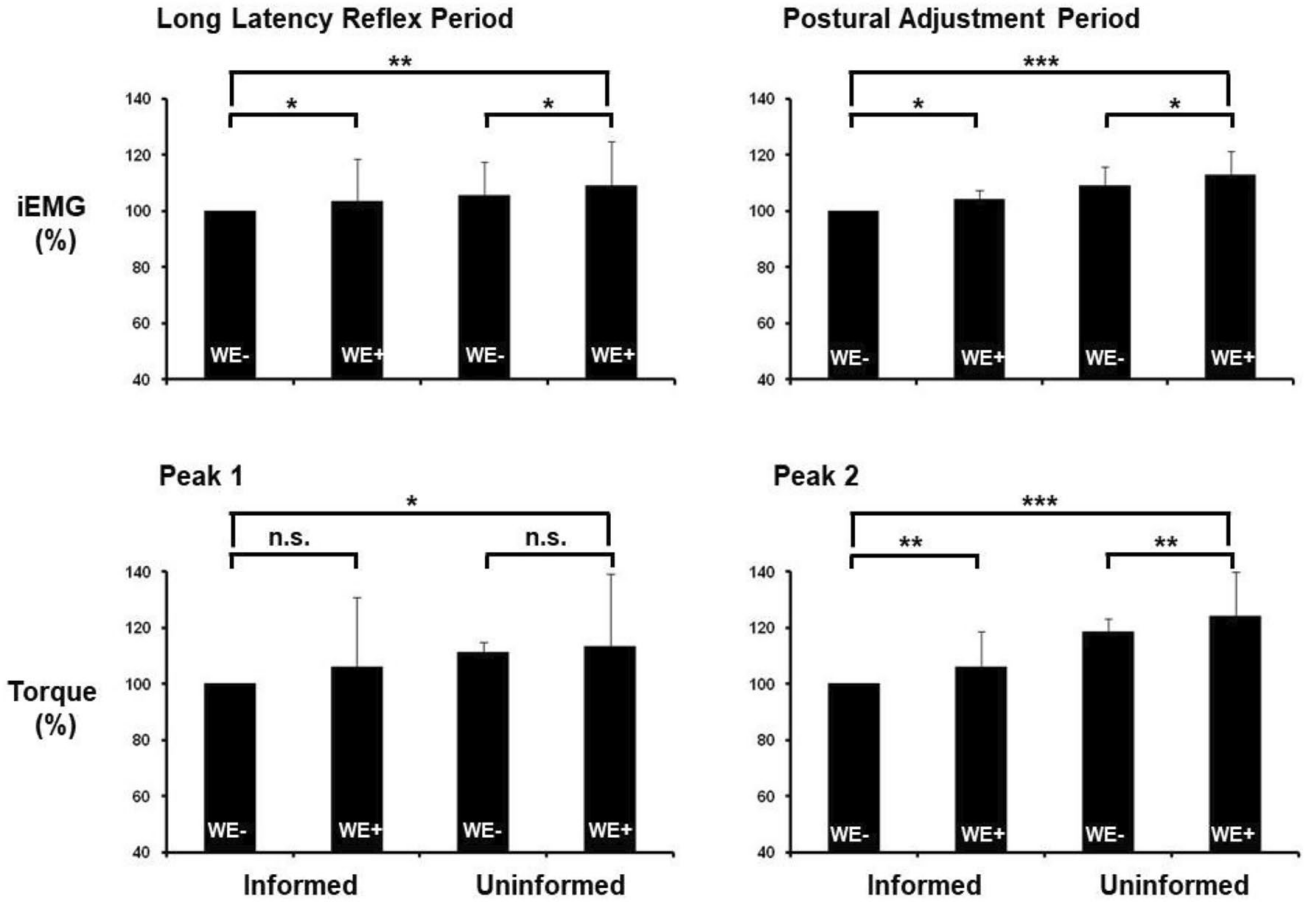
Amount of reactive muscular activity and reactive torque of the passively moved leg (240°/s knee flexion) according to task planning. All data are normalized to the condition *Informed-Rest*. The bars represent mean values (whiskers: one standard deviation) in the conditions *Informed-Rest, Informed-Reaction*, *Uninformed-Rest*, and *Uninformed-Reaction* (see text for detailed descriptions). Asterisks define the level of significance for group comparisons (*p < 0.05, **p < 0.01, ***p < 0.001)

However, *Uninformed* trials yielded larger torque amplitudes as compared to *Informed* trials (first peak: *F*_1,52_ = 3.8; *p* < 0.05; second peak: *F*_1,52_ = 15.5; *p* < 0.001), and *Reaction* trials resulted in larger torque amplitudes vs *Rest* trials for the second peak (*F*_1,52_ = 8.9; *p* < 0.01) without INFORMATION × TASK interaction (*F*_1,52_ = 3.5; *p* = 0.06) but not for the first peak (*F*_1,52_ = 0.7; *p* > 0.05) (Fig. 5). Influence of task planning is summarized in Fig. 6 and Table 1.

**Table 1:** Influence of task planning, i.e., how knowledge about upcoming IS velocity and preparation of a motor task in the upper limbs modulates reactions in rectus femoris muscle in 240°/s trials

	EMG in rectus femoris muscle			Torque in passively moved leg	
	SLR period (latency, area)	LLR period (area)	PA period (area)	Latencies	Amplitudes
*Uninformed vs. informed*	Similar	Larger	Larger	Similar first and second peak	Larger first and second peak
*Reaction vs. rest*	Similar	Larger	Larger	Similar first and second peak	Larger second peak

EMG: electromyography; SLR: short latency reflex; LLR: long latency reflex; PA: postural adjustment

According to the model of Mrachacz-Kersting and Sinkjaer (2003), the estimated postural and reflex components for these 240°/s trials normalized to the condition *Informed-Rest* (100 Nm) were, respectively: *Informed-Reaction* (108 ± 28 Nm; 113 ± 15 Nm), *Uninformed-Reaction* (111 ± 54 Nm; 123 ± 16 Nm), and *Uninformed-Rest* (135 ± 43 Nm; 120 ± 16 Nm).

### Startle modulation of performance

**Fig. 7:**
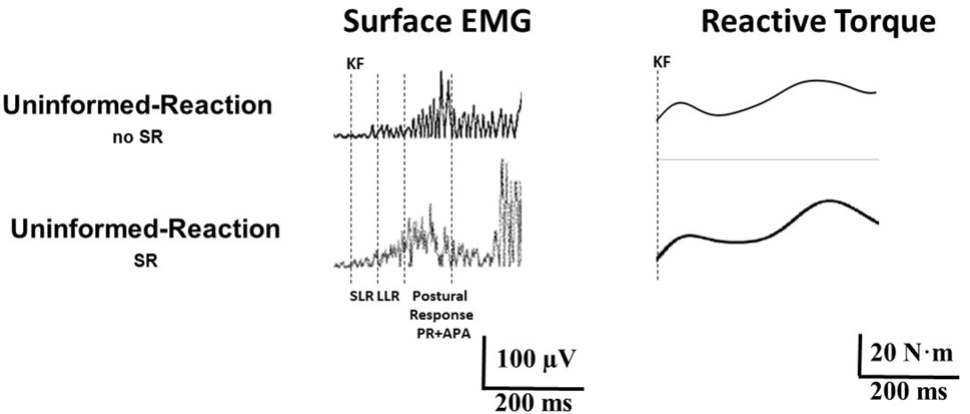
Startle modulation of performance. Surface EMG from rectus femoris muscle and reactive torques of the extended leg to knee flexion for representative 240°/s trials in the conditions *Uninformed-Reaction*_noSR_ *and Uninformed-Reaction*_SR_. The elicitation of a startle reaction resulted in larger APA and CPA activities and in a longer latency and higher amplitude of the first torque peak, and a later second torque peak. Acronyms as in Fig. 1

Startle reflexes and StartReact effects appeared only in some *Uninformed* trials at 240°/s velocity when subjects were highly prepared to react with a WE but were unaware about the upcoming IS velocity (*Uninformed-Reaction*_SR,_ *Uninformed-Reaction*_OPP-SR_ and *Uninformed-Reaction*_PP-SR_). As actual performance of leg movement (and associated reflexes and reactions) might be affected by such startle modulation, we compared ECR and RF responses in these three fast *Uninformed* conditions against *Uninformed-Reaction*_no SR_, i.e., without startle modulation, applying a one-factor ANOVA. A representative example is shown in Fig. 7.

EMG in ECR began significantly earlier in 240°/s trials containing startle modulation (*Uninformed-Reaction*_SR,_ *Uninformed-Reaction*_OPP-SR_, *Uninformed-Reaction*_PP-SR_) as compared to those without (*Uninformed-Reaction*, *Informed-Reaction*) with respective latencies of 206 ± 34 ms versus 247 ± 56 ms (*F*_6,91_ = 6.95; *p* < 0.05). This response acceleration concurs with a StartReact effect. Startle-related EMG in OOc and SCM occurred at 77 ± 25 ms and 85 ± 18 ms, respectively. Some trials showed late SCM activity at 181 ± 46 ms, presumably belonging to the WE motor program.

**Fig. 8:**
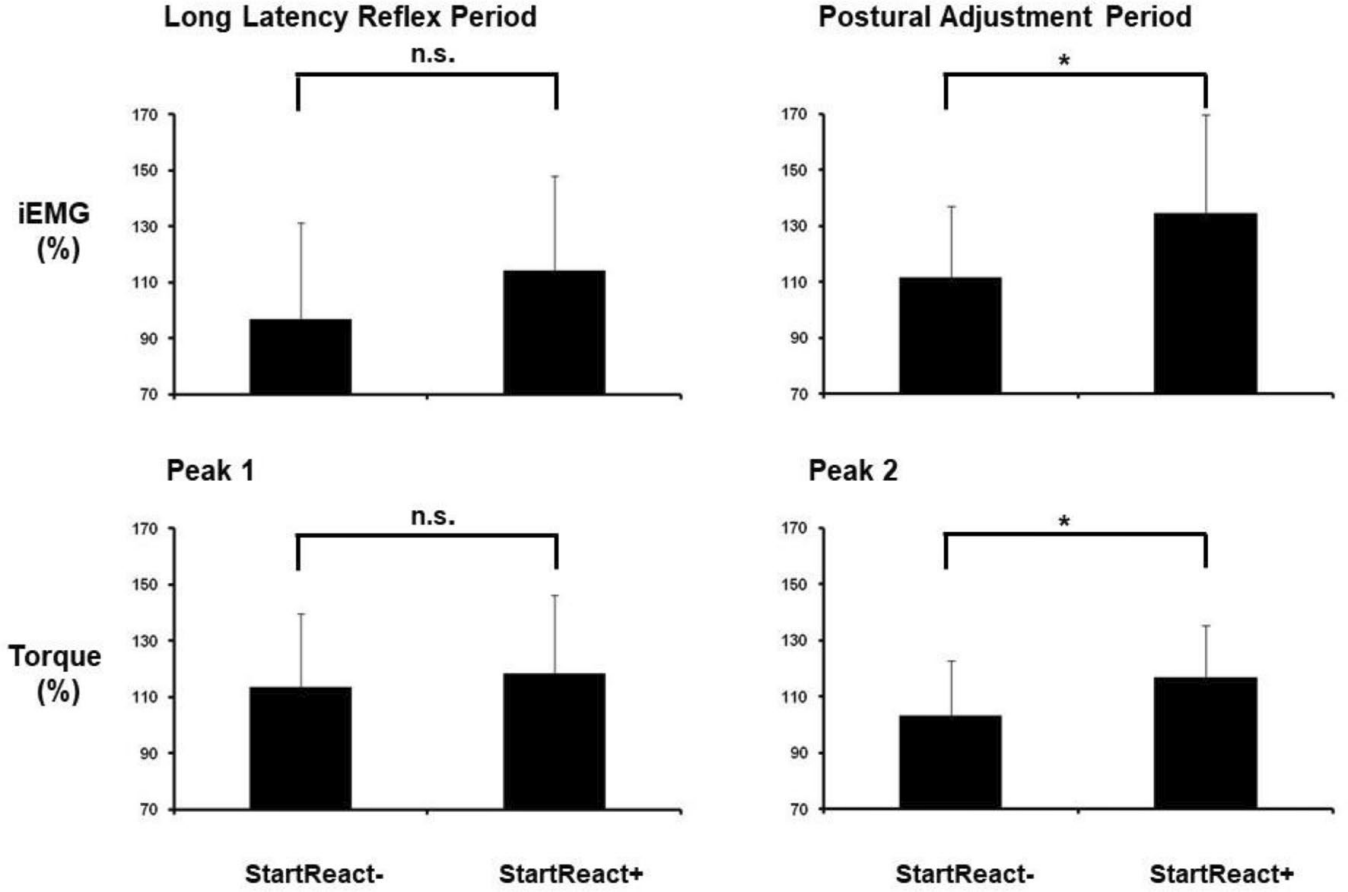
Amount of reactive muscular activity and reactive torque of the passively moved leg (240°/s knee flexion) in those conditions in which subjects were uninformed about the velocity of knee flexion and were required to perform a fast wrist extension. All data are normalized to the condition *Informed-Rest*. StartReact + denotes those conditions containing a StartReact effect in ECR (*Uninformed-Reaction*_SR,_ *Uninformed-Reaction*_OPP-SR_, *Uninformed-Reaction*_PP-SR_). StartReact- denotes those conditions without the effect (*Uninformed-Reaction*_noSR_). The bars represent mean values (whiskers: one standard deviation). Asterisks define the level of significance for group comparisons (*p < 0.05)

Concerning responses in RF, startle reflex presence did not significantly affect SLR latency (*F*_1,26_ = 0.01; *p* > 0.05), SLR area (*F*_1,26_ = 0.18; *p* > 0.05), nor LLR area (*F*_1,26_ = 2.71; *p* > 0.05), but resulted in larger PA area (*F*_1,26_ = 4.51; *p* < 0.05) in *Uninformed* trials with startle modulation compared to those without (Fig. 8).

In torque recordings, the presence of a startle reaction tended to prolong the latency (F_1,26_ = 0.02; *p* > 0.05) and to augment the size (*F*_1,26_ = 0.01; *p* > 0.05) of the first torque peak, and to delay the second torque peak (*F*_1,26_ = 0.12; *p* > 0.05), although not reaching statistical significance. Startle signs presence resulted in a significantly larger second torque peak amplitude (*F*_1,26_ = 4.63; *p* < 0.05) as compared to 240°/s *Uninformed-Reaction*_no SR_ trials (Fig. 7). Influence of a startle reaction on task performance is summarized in Fig. 8 and Table 2.

**Table 2:** Influence of a startle reaction on task performance, i.e., how the presence of a startle, as seen by signs in orbicularis oculi and sternocleidomastoid muscles, modulates reactions in extensor carpi radialis (ECR) and rectus femoris muscles in 240°/s trials compared to corresponding trials without startle signs

	EMG in ECR	EMG in rectus femoris muscle			Torque in passively moved leg	
	Latency	SLR period (latency, area)	LLR period (area)	PA period (area)	Latencies	Amplitudes
*Uninformed-Reaction*_*no SR*_* vs Uninformed-Reaction*_SR_	Earlier	Similar	Similar	Larger	First and second peak delayed (n.s.)	Larger first peak (n.s.), larger second peak
*Uninformed-Reaction*_*no SR*_* vs Uninformed-Reaction*_OPP-SR_	Earlier	Similar	Similar	Larger	First and second peak delayed (n.s.)	Larger first peak (n.s.), larger second peak
*Uninformed-Reaction*_*no SR*_* vs Uninformed-Reaction*_PP-SR_	Earlier	Similar	Similar	Larger	First and second peak delayed (n.s.)	Larger first peak (n.s.), larger second peak

EMG: electromyography; SLR: short latency reflex; LLR: long latency reflex; PA: postural adjustment; n.s.: not significant

According to the model of Mrachacz-Kersting and Sinkjaer (2003), the estimated postural and reflex components for these *Uninformed* trials with startle modulation normalized to the condition *Informed-Rest* (100 Nm) were 117 ± 37 Nm and 112 ± 18 Nm, respectively.

## Discussion

### General remarks

The present study investigated how a subject’s response to a kinematic disturbance, which may compromise postural equilibrium, is modified by stimulus expectation and by pre-programming a motor task. The main difference to previous studies in the field is the particular positioning of the participants, who were suspended freely in the air without foot support, being secured by a harness. In previous studies, subjects were usually investigated standing upright. Thus for the requested tasks the fulcrum was located at the lower limbs, either at the ankle or the hip (Horak and Nashner 1986; Sherief et al. 2015; Ivanenko et al. 1997). Here, subjects were engaged in a situation where the legs may still contribute to posture, and thus postural adjustments, but not to sustaining body weight. In such a setting, leg responses depend on a subject’s preparedness for the upcoming stimulus and the specific task to be performed.

### Influence of speed gain scaling

IS intensity, in the present case angular velocity of passive KF, which was used as IS for the requested WE, has a significant influence on the presence and amount of muscular activity in RF at latencies corresponding to LLR and PA. In most 6°/s trials there was no SLR visible. Absence of such reflex activity has previously been described for upper and lower limb joints when muscle stretch was of insufficient intensity (Lamontagne et al. 1998; Rabita et al. 2005). However, in the LLR period and later, when also voluntary reactions may appear, EMG activity was significantly larger in 240°/s trials compared to most 6°/s and 60°/s trials. Beyond 100 ms following stimulus delivery, all three angular velocities gave rise to RF responses. These could correspond to prolonged PRs, also called “triggered reactions” (Crago et al. 1976; Manning et al. 2012), or to APAs in trials requiring WE (Santos et al. 2010b; Massion 1992; Bouisset and Zattara 1987).

When voluntary WE was required in response to perception of leg movement, ensuing SLR latencies in RF did not differ among the three angular velocities. LLR area was larger when the KF velocity was faster. With all three angular velocities, ECR activity was followed by CPA in RF with latencies around 300–400 ms post-IS, similar to other models (Memari et al. 2010; Santos et al. 2010b). Both EMG in ECR and CPA in RF appeared earlier in 240°/s and later in 6°/s trials, concurring with accelerated pre-programmed motor responses associated with an IS of high intensity (Pins and Bonnet 1996; Valls-Solé et al. 2012; Castellote and Valls-Solé 2019). In 6°/s and 60°/s trials, lower limb torques resulted in low amplitude curves without distinct peaks, whereas in 240°/s trials, a high-amplitude curve with two peaks was obtained, reflecting the different velocity-dependent patterns of RF activation. Such differential modulation of torques related to angular velocity has previously been described in other studies, which have explored quadriceps and tibialis anterior muscles (Ghori et al. 1995; Nicol et al. 2003).

Muscular response and resulting torque may also depend on background EMG activity of the stretched muscle (Scheirs and Brunia 1986; Ogiso et al. 2002). In the present study, however, there was no significant difference in background EMG for both ECR and RF, thus not influencing the current results. The obtained torques consistently reflected the underlying muscular activity, and, as there was a long time gap between consecutive trials, they were likely not influenced by thixotropy from preceding trials, as previously discussed (Lamontagne et al. 1998).

### Influence of task planning

EMG activity obtained at latencies exceeding the LLR period deserves special attention, as it varied according to IS and task. With an acoustic IS, WE did not induce APAs in RF when subjects were suspended in the air without leg support. This result contrasts the well-known APAs for an upper limb reaction while bearing weight on the lower limbs, concurring with a lesser role of the lower limbs for maintaining equilibrium when suspended in the air. However, at longer latencies, WE required ensuing CPA in the legs, presumably as part of the motor program used by the brain to ensure balance (Alexandrov et al. 2005; Park et al. 2004; Santos et al. 2010b).

In the present study, two distinct motor program components must be considered in the legs: (1) PRs to passive leg movement, and (2) APAs required for the upper limb task. Both motor program components may overlap in time (Nashner and Cordo 1981), and both serve to rebalance posture taking into account that a large lower limb segment and a hand are moved simultaneously. RF responses following the LLR period may either correspond to a PR following leg disturbance (Horak et al. 1997; Diener et al. 1988) or an APA associated with the required WE task as previously described for upper limb movements (Belen’kiĭ et al. 1967; Woollacott and Manchester 1993; Bleuse et al. 2006; Yaguchi et al. 2017) and for displacements of lower limbs (Santos et al. 2010b; Massion 1992; Bouisset and Zattara 1987). The net result induced by stretching the RF is a consistent reactive torque, which is proportional to the angular velocity. The fastest stretch in RF also elicited a StartReact effect in the upper limb, resulting in accelerated WE.

In order to unravel the two motor program components, we first confirmed similar background EMG activity among conditions, as this is known to influence the resulting muscular activity (Scheirs and Brunia 1986; Ogiso et al. 2002). Then, we explored the presence of APAs, as well as CPAs associated with WE in this particular situation of being suspended without foot support and found only CPAs with a latency of some 130 ms following ECR activity but no preceding APAs. These findings suggest that WE per se is not such a strong movement requiring advance preparation involving RF. In fact, without ground contact, upright posture does not imply an inverse pendulum where an arm movement is preceded by activity aiming at stabilizing leg muscles (Stamenkovic et al. 2021; Berret et al. 2009). The presence of CPAs concurs with some disequilibrium induced by WE, which requires compensatory activity incorporating RF as described in other postural paradigms (Memari et al. 2010; Santos et al. 2010b).

### Startle modulation of performance

Some *Uninformed* trials performed at 240°/s angular velocity (i.e., high IS intensity) and requiring WE (i.e., preparation of a motor task) contained EMG activity in OOc or SCM, concurring with a startle reflex, and presented with acceleration and enhancement of EMG in ECR, consistent with a StartReact effect. This observation of an obvious change in overall response characteristics prompted us to re-analyze the respective data obtained in *Uninformed* trials after categorizing them according to presence/absence of startle-related activity in order to unravel possible differences in postural reactions.

Startle signs were not present in *Uninformed* 6°/s and 60°/s trials, indicating that stimuli, although unexpected, were not of sufficient intensity to activate the startle circuits located in the brainstem (Valls‐Solé et al. 1999; Valls-Solé et al. 2008; Maslovat et al. 2021). In contrast, 240°/s trials were obviously fast enough to induce occasional kinematic startles. The StartReact effect induced by fast leg movement, i.e., how the somatosensory stimulus modifies voluntary upper limb motor responses in a velocity-dependent manner, has previously been reported (Castellote et al. 2017). These findings are in agreement with other startling stimuli previously described (Carlsen et al. 2004; Castellote et al. 2012; Castellote and Kofler 2018).

When subjects were *Informed* about the upcoming IS intensity and when they were asked not to react with WE (*Rest*), no EMG activity was present in RF during the PA period. When a strong WE was required (*Reaction*), the same fast leg disturbance, however, resulted in EMG activity in RF in the PA period, which could correspond to either one of the two mentioned program components: APA (program associated with upper limb activity) or PR (program of lower limb postural reactions). Yet similar EMG activity was seen in RF during the PA period, induced by the same fast leg disturbance, when subjects were unaware of IS intensity (*Uninformed*) and without motor task (*Rest*), concurring with a PR, but larger when subjects were asked to perform WE (*Uninformed, React*): this concurs with a PR plus APA, as WE per se did not require anticipatory activity when triggered by an acoustic IS.

Possibly, a fast leg disturbance might occasionally have been startling when two conditions were simultaneously met: unknown IS velocity (*Uninformed*) and preparedness for a motor task when WE was required (*React*). Consequently, a StartReact effect was seen in ECR, and more importantly, EMG activity was significantly larger in RF in trials with startle signs. Such higher activity does not necessarily signify a postural challenge because larger EMG activity may indeed be seen due to startle reaction, mainly in muscles involved in voluntary activity (Valls‐Solé et al. 1999; Rothwell 2006; Carlsen et al. 2012). Startle reactions have rarely been described in postural challenges (Campbell et al. 2012), where they are not always present (Nonnekes et al. 2013).

Generally, a StartReact effect results in earlier and larger pre-programmed voluntary movements (DeLuca et al. 2022; McInnes et al. 2021). With the present paradigm, we previously reported significant acceleration of voluntary WE whenever startle reflexes were present in OOc and SCM (Castellote et al. 2017), even when a prepulse suppressed startle-related EMG activity in the latter muscles (Valls-Solé et al. 2005; Kumru et al. 2006). Concerning the lower limbs in the present study, startle reactions exerted no influence on the SLR in RF, which is known to be only spinally modulated and not affected by startle stimuli (Shemmell 2015). The absence of an influence of startle stimuli on EMG activity in RF during the LLR period suggests that the respective RF activity is part of a reflex and not a consequence of voluntary drive. In contrast, larger EMG activity in the PA period, accompanied by larger torque amplitudes in the second torque peak, may correspond to an APA related to WE, augmented by the startle, as both APA and WE are part of the same motor program. In fact, EMG activity in ECR was accelerated and larger in startle trials (Castellote et al. 2017), which is entirely coherent with a larger APA in RF, together with larger peak torque, in the same trials.

Based on previously published models (Mrachacz-Kersting and Sinkjaer 2003), the estimated reflex torque component did not change relative to the condition *Informed-Rest*, which is coherent with no modification of reflex responses by the startling stimulus. However, the postural component was larger in the context of a StartReact effect, consistent with a common voluntary motor program of WE plus APA.

Indeed, the actual motor program depends on the type of IS, acoustic or kinematic, the advance knowledge about the upcoming IS intensity, here angular velocity of KF, and the known requirement of a motor task, here WE. When a fast WE is required and KF velocity is known, the leg responds with an APA, which is not present when the IS is a known non-startling sound. When the subject is not informed about the upcoming KF velocity, WE is accompanied in RF by a postural response that may include two components, one the consequence of leg displacement, the other one due to the fast wrist displacement. We hypothesize that when the subject is informed, hence in control of the situation, the brain prioritizes the task, thus an APA is the evident lower limb result. However, when the subject is not informed, hence not in full control of the situation, perhaps feeling some kind of threat, the brain gives priority to posture, thus the postural response appears—and an APA may be jointly delivered if needed. Thus, the postural response is prepared according to knowledge or uncertainty about the upcoming stimulus type and intensity.

For the time beyond, e.g., the LLR period and later, supraspinal structures also participate in postural responses (Pruszynski and Scott 2012; Kurtzer 2015). Larger EMG activity in RF in that time window is associated with more uncertainty about IS velocity, corresponding to higher amplitudes in first and second torque peaks. Although this response contains no voluntary elements, subjects may still unconsciously condition an optimal response execution. Expectancy of a stimulus may determine how supraspinal structures modulate reactive postural responses (Lim et al. 2017; Cesari et al. 2022; Dakin and Bolton 2018; Ritzmann et al. 2018; Kluft et al. 2020). With a StartReact response, the APA is delivered as part of the prepared motor program. Increased torque values of RF support this assumption: had the startle reaction been a “whole body response” to an unexpected stimulus, predominant flexor activity would be expected, thus antagonizing the extensor contraction in RF. One limitation of the present study is that flexor muscle EMG was not recorded due to technical limitations.

In addition to the presence of respective RF EMG activity in *Informed* (*React*) trials, consistent with APAs, and in *Uninformed* (*React*) trials, consistent with APAs plus PR, the presence of APAs within the StartReact response is further supported by the enhanced activity in *Uninformed-Reaction* trials containing startle signs, consistent with previous StartReact models (MacKinnon et al. 2007; Queralt et al. 2008).

### Postural motor program and wrist extension motor set

The influence of WE on the resulting “whole-body motor program” merits further comments. When in some trials a voluntary motor task is required, the subject prepares the motor set to optimize task performance. The resulting WE may challenge the subject’s postural equilibrium. On the low end, trials in block 1 (acoustic IS) revealed only postural compensation in RF after the WE, without affecting the SLR period and thus in line with no lower limb spinal cord involvement by the upper limb task. There was, however, EMG activity in RF during the LLR period and later. Passive KF only induced EMG activity in RF during the LLR and PA periods, in line with postural responses. More interestingly, when subjects had to prepare the WE motor set, concomitant RF activity was larger in all these periods. The present results are in line with previous reports concerning modulation of stretch reflexes and tendon jerks due to preparation of a voluntary motor response (Evarts and Tanji 1974; Forgaard et al. 2015).

Only scarce information, however, is available concerning postural responses, which seem to be at least in part task-dependent (Finley et al. 2013; Doemges and Rack 1992a, b; Dietz et al. 1994; Perreault et al. 2008; Shemmell et al. 2009; Krutky et al. 2010). This observation concurs with the findings in block 1, when subjects had to perform WE in the absence of passive KF (acoustic IS only), as there was no EMG activity in RF during these time periods, suggesting that some APAs appear only when the leg is moved in advance of WE. This is concordant with a significantly larger second torque peak (with its zenith preceding EMG in ECR and the associated PCs) than when the leg was not moved in advance. Consequently, the appropriate supraspinal structures that orchestrate the RF reaction in response to a fast passive stretch should integrate not only the postural response to the leg movement, but also the subsequent WE program that might as well be modulated by the passive RF elongation, because the same RF is part of a general postural program.

### Potential applications in rehabilitation settings

The main goal of the present study was to advance the current knowledge about motor preparation and the influence of a subject’s expectation and preparedness to perform an upper limb motor task on associated motor reactions in the legs. In addition, however, the present findings may serve to have some clinical relevance concerning neurorehabilitation. Nowadays, the empowering of patients seems to be a clinical fact in the recovery process (Sit et al. 2016; Hartford et al. 2019). Two main aspects in rehabilitation could benefit from the present results: assessment and therapy. This is the case for stroke patients placed in exoskeletons, in whom abnormal reflexes may interfere with supraspinal volitional commands. These subjects may require rehabilitation of a disrupted motor pattern that comprises reflexes, postural adjustments, and voluntary muscular activity. When assessing equilibrium in these patients, attention should be paid to modifications during the time windows of LLRs and PAs. If a patient is being assessed or treated applying a protocol that includes a fast leg flexion as stimulus, and if that perturbation is unexpected, or if the patient has to prepare a hand reaction, then the response in RF which is necessary for establishing or maintaining equilibrium should be larger in both time windows, whereas leg torque should be larger mainly for the first peak, due to task planning according to the present results. If these differences do not appear, it might be the case that the consequences of the patient’s stroke may prevent the implementation of an adequate motor program. If a patient's response includes signs of a StartReact effect, the reticulospinal tract can be considered to be preserved, as has been previously described in StartReact studies in stroke survivors (Carlsen et al. 2012; Honeycutt and Perreault 2012). In case that recordings of startle signs (in OOc and/or SCM) are not obtained, main signs to differentiate startle modulation of performance from task planning and preparedness could be an earlier latency of responses in ECR (i.e., a StartReact effect) and larger torque peaks with longer latencies in the leg, as described in the present study.

In conclusion, the present results show in detail the reactions of a leg muscle to passive displacement, which varies in different time periods, when the subject is engaged in the mental preparation for a motor task in the upper limb. The reaction depends mainly on whether the subject is informed or not about the nature of the upcoming IS, i.e., the passive flexion of the leg of interest, and may be additionally modulated by a startle stimulus, if the IS is strong and surprising enough in the context of movement preparation. In order to disentangle APAs from PRs, we investigated the subjects suspended in the air without foot support. The visible net result obtained from the EMG in RF when passive leg movement was the trigger to react, includes an APA as part of a motor program, which is not found with a known non-startling acoustic stimulus, and a PR. When the upcoming IS is known, i.e., KF velocity, WE requires the activation of leg extensors as part of the whole-body motor program, but when the subject is uninformed about the upcoming velocity of KF, the imposed uncertainty might cause an uneasy or even threatening situation (Lim et al. 2017; Cesari et al. 2022; Kluft et al. 2020), and the nervous system chooses a motor set that triggers PAs, in addition to APAs when a wrist reaction is required. And finally, when the subject is uninformed, and the intensity of the leg IS is strong enough to trigger a startle reaction, the APA becomes part of the StartReact response.

## Data Availability

For ethical reasons, the data are unsuitable for public deposition. Data supporting the present findings may be obtained from the corresponding author upon reasonable request.

## References

[CR1] Alexandrov Av, Frolov Aa, Horak Fb, Carlson-Kuhta P, Park S (2005). Feedback equilibrium control during human standing. Biol Cybern.

[CR2] Baker JM (2018). Gait disorders. Am J Med.

[CR3] Belen’kiĭ VE, Gurfinkel’ VS, Pal’tsev EI (1967). Control elements of voluntary movements. Biofizika.

[CR4] Bergmann J, Kramer A, Gruber M (2013). Repetitive hops induce postactivation potentiation in triceps surae as well as an increase in the jump height of subsequent maximal drop jumps. PLoS ONE.

[CR5] Bergui M, Lopiano L, Paglia G, Quattrocolo G, Scarzella L, Bergamasco B (1992). Stretch reflex of quadriceps femoris and its relation to rigidity in Parkinson’s disease. Acta Neurol Scand.

[CR6] Berret B, Bonnetblanc F, Papaxanthis C, Pozzo T (2009). Modular control of pointing beyond arm’s length. J Neurosci.

[CR7] Bleuse S, Cassim F, Blatt J-L, Labyt E, Derambure P, Guieu J-D, Defebvre L (2006). Effect of age on anticipatory postural adjustments in unilateral arm movement. Gait Posture.

[CR8] Bouisset S, Zattara M (1987). Biomechanical study of the programming of anticipatory postural adjustments associated with voluntary movement. J Biomech.

[CR9] Campbell AD, Chua R, Inglis JT, Carpenter MG (2012). Startle induces early initiation of classically conditioned postural responses. J Neurophysiol.

[CR10] Carlsen AN, Chua R, Inglis JT, Sanderson DJ, Franks IM (2004). Prepared movements are elicited early by startle. J Mot Behav.

[CR11] Carlsen AN, Dakin CJ, Chua R, Franks IM (2007). Startle produces early response latencies that are distinct from stimulus intensity effects. Exp Brain Res.

[CR12] Carlsen AN, Maslovat D, Lam MY, Chua R, Franks IM (2011). Considerations for the use of a startling acoustic stimulus in studies of motor preparation in humans. Neurosci Biobehav Rev.

[CR13] Carlsen AN, Maslovat D, Franks IM (2012). Preparation for voluntary movement in healthy and clinical populations: evidence from startle. Clin Neurophysiol.

[CR14] Castellote JM, Kofler M (2018). StartReact effects in first dorsal interosseous muscle are absent in a pinch task, but present when combined with elbow flexion. PLoS ONE.

[CR15] Castellote JM, Valls-Solé J (2019). Temporal relationship between perceptual and physiological events triggered by nociceptive heat stimuli. Sci Rep.

[CR16] Castellote JM, Kumru H, Queralt A, Valls-Solé J (2007). A startle speeds up the execution of externally guided saccades. Exp Brain Res.

[CR17] Castellote JM, Queralt A, Valls-Solé J (2012). Preparedness for landing after a self-initiated fall. J Neurophysiol.

[CR18] Castellote JM, Kofler M, Mayr A, Saltuari L (2017). Evidence for startle effects due to externally induced lower limb movements: implications in neurorehabilitation. Biomed Res Int.

[CR19] Cesari P, Piscitelli F, Pascucci F, Bertucco M (2022). Postural threat influences the coupling between anticipatory and compensatory postural adjustments in response to an external perturbation. Neuroscience.

[CR20] Crago PE, Houk JC, Hasan Z (1976). Regulatory actions of human stretch reflex. J Neurophysiol.

[CR21] da Costa CSN, Savelsbergh G, Rocha NACF (2010). Sit-to-stand movement in children: a review. J Mot Behav.

[CR22] Dakin CJ, Bolton DAE (2018). Forecast or fall: prediction’s importance to postural control. Front Neurol.

[CR23] Delafontaine A, Vialleron T, Hussein T, Yiou E, Honeine J-L, Colnaghi S (2019). Anticipatory postural adjustments during gait initiation in stroke patients. Front Neurol.

[CR24] DeLuca M, Low D, Kumari V, Parton A, Davis J, Mohagheghi AA (2022). A systematic review with meta-analysis of the StartReact effect on motor responses in stroke survivors and healthy individuals. J Neurophysiol.

[CR25] Diener HC, Horak FB, Nashner LM (1988). Influence of stimulus parameters on human postural responses. J Neurophysiol.

[CR26] Dietz V, Quintern J, Sillem M (1987). Stumbling reactions in man: significance of proprioceptive and pre-programmed mechanisms. J Physiol.

[CR27] Dietz V, Discher M, Trippel M (1994). Task-dependent modulation of short- and long-latency electromyographic responses in upper limb muscles. Electroencephalogr Clin Neurophysiol Evoked Potentials Section.

[CR28] Doemges F, Rack PM (1992). Changes in the stretch reflex of the human first dorsal interosseous muscle during different tasks. J Physiol.

[CR29] Doemges F, Rack PM (1992). Task-dependent changes in the response of human wrist joints to mechanical disturbance. J Physiol.

[CR30] Evarts EV, Tanji J (1974). Gating of motor cortex reflexes by prior instruction. Brain Res.

[CR31] Finley JM, Dhaher YY, Perreault EJ (2013). Acceleration dependence and task-specific modulation of short- and medium-latency reflexes in the ankle extensors. Physiol Rep.

[CR32] Fiset F, McFadyen BJ (2020). The switching of trailing limb anticipatory locomotor adjustments is uninfluenced by what the leading limb does, but general time constraints remain. Appl Sci.

[CR33] Forgaard CJ, Franks IM, Maslovat D, Chin L, Chua R (2015). Voluntary reaction time and long-latency reflex modulation. J Neurophysiol.

[CR34] Forgaard CJ, Franks IM, Bennett K, Maslovat D, Chua R (2018). Mechanical perturbations can elicit triggered reactions in the absence of a startle response. Exp Brain Res.

[CR35] Garcia-Rill E, Saper CB, Rye DB, Kofler M, Nonnekes J, Lozano A, Valls-Solé J, Hallett M (2019). Focus on the pedunculopontine nucleus. Consensus review from the May 2018 brainstem society meeting in Washington, DC, USA. Clin Neurophysiol.

[CR36] Ghori GMU, Donne B, Luckwill RG (1995). Relationship between torque and EMG activity of a knee extensor muscle during isokinetic concentric and eccentric actions. J Electromyogr Kinesiol.

[CR37] Hartford W, Lear S, Nimmon L (2019). Stroke survivors’ experiences of team support along their recovery continuum. BMC Health Serv Res.

[CR38] Helm M, Ritzmann R, Gollhofer A, Freyler K (2019). Anticipation modulates neuromechanics of drop jumps in known or unknown ground stiffness. PLoS ONE.

[CR39] Honeycutt CF, Perreault EJ (2012). Planning of ballistic movement following stroke: insights from the startle reflex. PLoS ONE.

[CR40] Horak FB, Nashner LM (1986). Central programming of postural movements: adaptation to altered support-surface configurations. J Neurophysiol.

[CR41] Horak FB, Diener HC, Nashner LM (1989). Influence of central set on human postural responses. J Neurophysiol.

[CR42] Horak FB, Henry SM, Shumway-Cook A (1997). Postural perturbations: new insights for treatment of balance disorders. Phys Ther.

[CR43] Ivanenko YP, Levik YS, Talis VL, Gurfinkel VS (1997). Human equilibrium on unstable support: the importance of feet-support interaction. Neurosci Lett.

[CR44] Jacobs JV, Horak FB (2007). Cortical control of postural responses. J Neural Transm.

[CR45] Kluft N, Bruijn SM, Luu MJ, van Dieën JH, Carpenter MG, Pijnappels M (2020). The influence of postural threat on strategy selection in a stepping-down paradigm. Sci Rep.

[CR46] Krutky MA, Ravichandran VJ, Trumbower RD, Perreault EJ (2010). Interactions between limb and environmental mechanics influence stretch reflex sensitivity in the human arm. J Neurophysiol.

[CR47] Kumru, H., Valls-Solé, J., Kofler, M., Castellote, J., & Sanegre, T. (2006). Chapter 9 The effects of a prepulse on the StartReact phenomenon. En *Supplements to Clinical Neurophysiology* (Vol. 58, pp. 101–109). Elsevier. 10.1016/S1567-424X(09)70062-5 10.1016/s1567-424x(09)70062-5 16623325

[CR48] Kurtzer IL (2015). Long-latency reflexes account for limb biomechanics through several supraspinal pathways. Front Integr Neurosci.

[CR49] Lamontagne A, Malouin F, Richards CL, Dumas F (1998). Evaluation of reflex- and nonreflex-induced muscle resistance to stretch in adults with spinal cord injury using hand-held and isokinetic dynamometry. Phys Ther.

[CR50] Lee RG, Tatton WG (1975). Motor responses to sudden limb displacements in primates with specific CNS lesions and in human patients with motor system disorders. Can J Neurol Sci.

[CR51] Leukel C, Lundbye-Jensen J, Gruber M, Zuur AT, Gollhofer A, Taube W (2009). Short-term pressure induced suppression of the short-latency response: a new methodology for investigating stretch reflexes. J Appl Physiol.

[CR52] Liaw J-W, Chen R-S, Chen VC-F, Wang Y-R, Chan H-L, Chang Y-J (2021). Evaluation of anticipatory postural adjustment before quantified weight shifting—system development and reliability test. Appl Sci.

[CR53] Lim SB, Cleworth TW, Horslen BC, Blouin J-S, Inglis JT, Carpenter MG (2017). Postural threat influences vestibular-evoked muscular responses. J Neurophysiol.

[CR54] MacKinnon CD, Bissig D, Chiusano J, Miller E, Rudnick L, Jager C, Zhang Y, Mille M-L, Rogers MW (2007). Preparation of anticipatory postural adjustments prior to stepping. J Neurophysiol.

[CR55] Manning CD, Tolhurst SA, Bawa P (2012). Proprioceptive reaction times and long-latency reflexes in humans. Exp Brain Res.

[CR56] Maslovat D, Carter MJ, Kennefick M, Carlsen AN (2014). Startle neural activity is additive with normal cortical initiation-related activation. Neurosci Lett.

[CR57] Maslovat D, Sadler CM, Smith V, Bui A, Carlsen AN (2021). Response triggering by an acoustic stimulus increases with stimulus intensity and is best predicted by startle reflex activation. Sci Rep.

[CR58] Massion J (1992). Movement, posture and equilibrium: interaction and coordination. Prog Neurobiol.

[CR59] Massion J, Ioffe M, Schmitz C, Viallet F, Gantcheva R (1999). Acquisition of anticipatory postural adjustments in a bimanual load-lifting task: normal and pathological aspects. Exp Brain Res.

[CR60] Mayr A, Kofler M, Quirbach E, Matzak H, Fröhlich K, Saltuari L (2007). Prospective, blinded, randomized crossover study of gait rehabilitation in stroke patients using the lokomat gait orthosis. Neurorehabil Neural Repair.

[CR61] Mayr A, Quirbach E, Picelli A, Kofler M, Smania N, Saltuari L (2019). Early robot-assisted gait retraining in non-ambulatory patients with stroke: a single blind randomized controlled trial. Eur J Phys Rehab Med.

[CR62] McInnes AN, Castellote JM, Kofler M, Honeycutt CF, Lipp OV, Riek S, Tresilian JR, Marinovic W (2021). Cumulative distribution functions: an alternative approach to examine the triggering of prepared motor actions in the StartReact effect. Eur J Neurosci.

[CR63] McPherson JG, Stienen AH, Drogos JM, Dewald JP (2018). Modification of spastic stretch reflexes at the elbow by flexion synergy expression in individuals with chronic hemiparetic stroke. Arch Phys Med Rehabil.

[CR64] Memari S, Bouisset S, Le Bozec S (2010). Consecutive postural adjustments in a single step. Comput Methods Biomech Biomed Engin.

[CR65] Mirbagheri MM, Niu X, Kindig M, Varoqui D (2012). The effects of locomotor training with a robotic-gait orthosis (Lokomat) on neuromuscular properties in persons with chronic SCI. Annual Int Conf IEEE Eng Med Biol Soc.

[CR66] Morris ME, Huxham F, McGinley J, Dodd K, Iansek R (2001). The biomechanics and motor control of gait in Parkinson disease. Clin Biomech.

[CR67] Mrachacz-Kersting N, Sinkjaer T (2003). Reflex and non-reflex torque responses to stretch of the human knee extensors. Exp Brain Res.

[CR68] Mrachacz-Kersting N, Lavoie BA, Andersen JB, Sinkjaer T (2004). Characterisation of the quadriceps stretch reflex during the transition from swing to stance phase of human walking. Exp Brain Res.

[CR69] Mrachacz-Kersting N, Grey MJ, Sinkjær T (2006). Evidence for a supraspinal contribution to the human quadriceps long-latency stretch reflex. Exp Brain Res.

[CR70] Nashner LM, Cordo PJ (1981). Relation of automatic postural responses and reaction-time voluntary movements of human leg muscles. Exp Brain Res.

[CR71] Nicol C, Kuitunen S, Kyröläinen H, Avela J, Komi PV (2003). Effects of long- and short-term fatiguing stretch-shortening cycle exercises on reflex EMG and force of the tendon-muscle complex. Eur J Appl Physiol.

[CR72] Nonnekes J, Scotti A, Oude Nijhuis LB, Smulders K, Queralt A, Geurts ACH, Bloem BR, Weerdesteyn V (2013). Are postural responses to backward and forward perturbations processed by different neural circuits?. Neuroscience.

[CR73] Ogiso K, McBride JM, Finni T, Komi PV (2002). Short-latency stretch reflex modulation in response to varying soleus muscle activities. J Electromyogr Kinesiol.

[CR74] Park S, Horak FB, Kuo AD (2004). Postural feedback responses scale with biomechanical constraints in human standing. Exp Brain Res.

[CR75] Perreault EJ, Chen K, Trumbower RD, Lewis G (2008). Interactions with compliant loads alter stretch reflex gains but not intermuscular coordination. J Neurophysiol.

[CR76] Pins D, Bonnet C (1996). On the relation between stimulus intensity and processing time: Piéron’s law and choice reaction time. Percept Psychophys.

[CR77] Pruszynski JA, Scott SH (2012). Optimal feedback control and the long-latency stretch response. Exp Brain Res.

[CR78] Pruszynski JA, Kurtzer I, Scott SH (2008). Rapid motor responses are appropriately tuned to the metrics of a visuospatial task. J Neurophysiol.

[CR79] Queralt A, Valls-Solé J, Castellote JM (2008). The effects of a startle on the sit-to-stand manoeuvre. Exp Brain Res.

[CR80] Rabita G, Dupont L, Thevenon A, Lensel-Corbeil G, Pérot C, Vanvelcenaher J (2005). Quantitative assessment of the velocity-dependent increase in resistance to passive stretch in spastic plantarflexors. Clin Biomech.

[CR81] Reissner L, Fischer G, List R, Giovanoli P, Calcagni M (2019). Assessment of hand function during activities of daily living using motion tracking cameras: a systematic review. Proc Inst Mech Eng [h].

[CR82] Ritzmann R, Lee K, Krause A, Gollhofer A, Freyler K (2018). Stimulus prediction and postural reaction: phase-specific modulation of soleus h-reflexes is related to changes in joint kinematics and segmental strategy in perturbed upright stance. Front Integr Neurosci.

[CR83] Rothwell, J. C. (2006). Chapter 18 The startle reflex, voluntary movement, and the reticulospinal tract. En Supplements to Clinical Neurophysiology (Vol. 58, pp. 223–231). Elsevier. 10.1016/S1567-424X(09)70071-6 10.1016/s1567-424x(09)70071-6 16623334

[CR84] Santello M, Bianchi M, Gabiccini M, Ricciardi E, Salvietti G, Prattichizzo D, Ernst M, Moscatelli A, Jörntell H, Kappers AML, Kyriakopoulos K, Albu-Schäffer A, Castellini C, Bicchi A (2016). Hand synergies: Integration of robotics and neuroscience for understanding the control of biological and artificial hands. Phys Life Rev.

[CR85] Santos MJ, Kanekar N, Aruin AS (2010). The role of anticipatory postural adjustments in compensatory control of posture: 1. Electromyographic analysis. J Electromyogr Kinesiol.

[CR86] Santos MJ, Kanekar N, Aruin AS (2010). The role of anticipatory postural adjustments in compensatory control of posture: 2. Biomechanical analysis. J Electromyogr Kinesiol.

[CR87] Scheirs JGM, Brunia CHM (1986). Motor preparation and the achilles tendon reflex: the role of background muscle tension. Biol Psychol.

[CR88] Shemmell J (2015). Interactions between stretch and startle reflexes produce task-appropriate rapid postural reactions. Front Integr Neurosci.

[CR89] Shemmell J, An JH, Perreault EJ (2009). The differential role of motor cortex in stretch reflex modulation induced by changes in environmental mechanics and verbal instruction. J Neurosci.

[CR90] Sherief AEAA, Abo Gazya AA, Abd El Gafaar MA (2015). Integrated effect of treadmill training combined with dynamic ankle foot orthosis on balance in children with hemiplegic cerebral palsy. Egypt J Med Hum Genet.

[CR91] Sit JW, Chair SY, Choi K, Chan CW, Lee DT, Chan AW, Cheung JL, Tang SW, Chan PS, Taylor-Piliae RE (2016). Do empowered stroke patients perform better at self-management and functional recovery after a stroke? A randomized controlled trial. Clin Interv Aging.

[CR92] Stamenkovic A, Hollands MA, Stapley PJ (2021). Constancy of preparatory postural adjustments for reaching to virtual targets across different postural configurations. Neuroscience.

[CR93] Vaidya T, Chambellan A, de Bisschop C (2017). Sit-to-stand tests for COPD: a literature review. Respir Med.

[CR94] Valls-Solé J, Rothwell JC, Goulart F, Cossu G, Muñoz E (1999). Patterned ballistic movements triggered by a startle in healthy humans. J Physiol.

[CR95] Valls-Solé J, Kofler M, Kumru H, Castellote JM, Sanegre MT (2005). Startle-induced reaction time shortening is not modified by prepulse inhibition. Exp Brain Res.

[CR96] Valls-Solé J, Kumru H, Kofler M (2008). Interaction between startle and voluntary reactions in humans. Exp Brain Res.

[CR97] Valls-Solé J, Castellote JM, Kofler M, Casanova-Molla J, Kumru H, Schestatsky P (2012). Awareness of temperature and pain sensation. J Pain.

[CR98] Valls-Solé J, Castellote JM, Kofler M, Serranová T, Versace V, Campostrini S, Campolo M (2021). When reflex reactions oppose voluntary commands: The StartReact effect on eye opening. Psychophysiology.

[CR99] Vedula S, Kearney RE, Wagner R, Stapley PJ (2010). Decoupling of stretch reflex and background muscle activity during anticipatory postural adjustments in humans. Exp Brain Res.

[CR100] Woollacott MH, Manchester DL (1993). Anticipatory postural adjustments in older adults: are changes in response characteristics due to changes in strategy?. J Gerontol.

[CR101] Yaguchi C, Fujiwara K, Kiyota N (2017). Activation timing of postural muscles of lower legs and prediction of postural disturbance during bilateral arm flexion in older adults. J Physiol Anthropol.

